# Predictors of Poor Outcomes in Chronic Obstructive Pulmonary Disease (COPD) Patients Admitted to the Emergency Department With COVID-19: A Prospective Study

**DOI:** 10.7759/cureus.71154

**Published:** 2024-10-09

**Authors:** Merve Osoydan Satici, Celal Satıcı, Mehmet Muzaffer İslam, İbrahim Altunok, Şeyma Başlılar, Sevde N Emir, Gökhan Aksel, Serkan Emre Eroğlu

**Affiliations:** 1 Emergency Medicine, Şişli Hamidiye Etfal Research and Training Hospital, Istanbul, TUR; 2 Pulmonology, Yedikule Chest Disease and Chest Surgery Training and Research Hospital, Istanbul, TUR; 3 Emergency Medicine, Ümraniye Training and Research Hospital, Istanbul, TUR; 4 Pulmonology, Ümraniye Training and Research Hospital, Istanbul, TUR; 5 Radiology, Ümraniye Training and Research Hospital, Istanbul, TUR

**Keywords:** copd: chronic obstructive pulmonary disease, covid-19, poor outcomes, pulmonary function test, radiological severity score, respiratory capacity, rox index

## Abstract

Objectives

Long-term consequences of COVID-19 in the post-pandemic era are still being investigated. Despite the growing data on COVID-19, there remains a lack of information regarding predictors of poor outcomes among chronic obstructive pulmonary disease (COPD) patients with COVID-19.

Methods

A single-center prospective cohort study was conducted with a total of 172 adult COPD patients with COVID-19 pneumonia included. Univariable and multivariable analyses were conducted to define independent factors associated with ICU admission, need for mechanical ventilation, or all-cause mortality in 30 days following COVID-19 pneumonia. receiver operating characteristic (ROC) analyses evaluated the diagnostic performance of the independent predictors.

Results

Out of all the patients, 73 (42.4%) experienced poor outcomes. Lower forced expiratory volume in the first second (FEV1) (OR= 0.949, p= 0.004), higher radiological severity score (OR= 1.15, p= 0.004), and lower respiratory rate oxygenation (ROX) index (OR= 0.867, p<0.001) were independently associated with poor outcomes. ROX index was found a better predictor of poor outcome than oxygen saturation (SaO2)/fraction of inspired oxygen (FiO2) and partial pressure of oxygen (PaO2)/FiO2 ratio (area under the curve (AUC)=0.80 vs. 0.73, p=0.01; AUC= 0. 80 vs. 0.63 p=0.001). A significant decline in FEV1 values compared to baseline values was observed (55.9 ±12.9 vs. 62.1±10.0; p<0.001).

Conclusion

Lower baseline FEV1, higher COVID-19 radiological severity score, and lower ROX index are strongly associated with poor outcomes in COPD patients with COVID-19.

## Introduction

COVID-19 primarily manifests in the respiratory tract, leading to detrimental consequences such as lung injury and the development of acute respiratory distress syndrome (ARDS) which represents the prevailing cause of mortality associated with the disease [1]. Identifying the risk factors associated with poor prognosis and mortality in COVID-19 is crucial as it allows early detection, appropriate monitoring, optimized treatment strategies, and the efficient allocation of medical resources [2].

Patients with chronic respiratory conditions, particularly chronic obstructive pulmonary disease (COPD), are at the forefront of the risk group for severe acute respiratory syndrome coronavirus 2 (SARS-CoV-2) infection due to diminished pulmonary reserve and increased expression of the angiotensin-converting enzyme 2 (ACE-2) receptor within the small airways [3]. Notably, viral infections are recognized as the prevailing cause of COPD exacerbations, thereby emphasizing the significance of COVID-19 pneumonia within this specific patient subgroup. Despite acknowledging the independent association between COPD and increased risks of hospitalization, severe illness, and mortality in the context of COVID-19 [4,5], data are insufficient in predicting poor outcomes among COPD patients with COVID-19 pneumonia. Given that COPD is the third leading cause of mortality on a global scale [6] and considering the increased vulnerability of this patient population, it becomes paramount to determine the mortality risks associated with COVID-19 in COPD patients.

Although there is a wealth of literature on COVID-19 infection, there remains notable scarce data regarding the long-term effects of COVID-19 in patients with chronic respiratory diseases, particularly in the context of COPD. It has been observed that even in COVID-19 patients without pre-existing pulmonary conditions, reduced lung function and radiological abnormalities can persist for up to six months following discharge [7]. It was revealed that 25.5% of patients with COVID-19-related lung involvement experienced persistent lung dysfunction three months after discharge, with 21% still exhibiting these abnormalities even six months after symptom onset [8]. In the case of COPD patients, the existing lung injury is accompanied by aberrant remodeling processes. Consequently, it is anticipated that substantial fibrosis may arise following SARS-CoV-2 infection, raising concerns regarding the trajectory of lung injury and the potential occurrence of permanent functional impairment.

Based on the aforementioned information, this study aimed to primarily investigate the parameters predicting poor outcomes in COPD patients admitted to the emergency department (ED) with COVID-19. Additionally, the secondary outcomes of the study were to assess changes in respiratory function among COPD patients during the post-COVID-19 period and to examine the factors associated with re-admission to the ED due to respiratory symptoms.

## Materials and methods

Study design and time period

This was an observational, prospective cohort study conducted at the Emergency Medicine Department of Ümraniye Training and Research Hospital, Istanbul, Türkiye, which handles approximately 500,000 admissions annually. The study was approved by the Clinical Trials Ethics Board Committee of the Ümraniye Training and Research Hospital (approval number: B.10.1.TKH.4.34.H.GP.0.01/343, dated November 24, 2020). Written informed consent was obtained from the patients participating in the study.

Study population

All adult patients admitted to the ED between December 1, 2020, and December 1, 2021, were assessed for inclusion in the study. We included patients with COPD who were diagnosed by a pulmonologist, confirmed to have COVID-19 through SARS-CoV-2 reverse transcription-polymerase chain reaction (RT-PCR) testing, and were willing to participate voluntarily. Conversely, patients under the age of 18, pregnant individuals, those lost to follow-up, and those lacking a pulmonary function test (PFT) conducted within the past year were excluded from the study.

Sample size

To determine the sample size, we utilized the sample size formula recommended by Green et al. [9] which is commonly used in studies where the primary outcome involves multivariate regression analysis. Based on previous research findings, the study aimed to identify a maximum of 10 potential variables that could predict poor outcomes. The sample size required to establish a possible model using multivariate logistic regression analysis was calculated using the formula N=50 + 8(k), considering a medium effect size (R2: 0.7: 0.20). To maintain a margin of error of 10%, an estimated sample size of 143 patients was required for our study.

Study setting

A thorough evaluation was conducted upon admission from eligible patients. This included assessing cigarette smoking history, occupational exposure, long-term oxygen therapy (LTOT) use, COVID-19 vaccination status, existing comorbidities, and recent PFT results performed within the last one year.

Standardization of vital parameters was achieved by employing a bedside monitor (BSM-4111K; Nihon Kohden Corporation, Shinjuku City, Tokyo, Japan) to measure pulse rate, blood pressure, and peripheral oxygen saturation (SpO2) values in all patients. Additionally, fever and respiratory rate were recorded. To estimate the fraction of inspired oxygen (FiO2%) in patients receiving oxygen therapy via nasal cannula or face mask, the formula FiO2 = 4 x (oxygen flow L/minute) + 21 was utilized, while a value of 21% was assumed for patients breathing room air [10]. For patients receiving noninvasive ventilation support, the FiO2 value set in the ventilator was taken into consideration. The respiratory rate oxygenation (ROX) index, a calculated value obtained by dividing the SpO2/FiO2 ratio by respiratory rate, was also utilized as a measure to evaluate the oxygenation status.

Patient management was carried out by experienced emergency medicine specialists. SARS-CoV-2 RT-PCR testing, arterial blood gas (ABG) analysis, complete blood count (CBC), biochemical profile tests and, if deemed necessary, lung imaging were performed. The need for hospitalization, intensive care unit (ICU) admission or mechanical ventilation, and treatment protocols for patients diagnosed with COVID-19 were determined by physicians evaluating the patients, in line with the guidelines provided by the Ministry of Health, Türkiye, during the study period [11]. 

Following their initial presentation to the ED, patients were subjected to a follow-up period of 30 days. COPD patients who did not experience poor outcomes were invited for a routine outpatient follow-up at the Chest Diseases clinic after three months, specifically for the assessment of pulmonary function. During this visit, PFTs were conducted, and the results were documented. The recorded forced expiratory volume in one second (FEV1) at admission was compared to the FEV1 values obtained during this third-month follow-up visit.

The Global Initiative for Chronic Obstructive Lung Disease (GOLD) staging of the patients was performed according to the 2022 guidelines: Stage 1 if FEV1 > 80%, Stage 2 if FEV1 is between 50-80%, Stage 3 if FEV1 is between 30-50%, and Stage 4 if FEV1 is < 30% [12]. A poor outcome was defined as admission to the ICU or mortality.

Radiological scoring

Scoring systems were utilized to evaluate radiologic parameters that could predict poor prognosis in COPD patients with COVID-19. The non-contrast thoracic computer tomography (CT) images of the patients enrolled in the study were carefully evaluated by a radiologist possessing clinical expertise, and the corresponding scores were calculated. The measurement of total lung capacity (TLC) using thoracic CT imaging provides valuable information regarding pulmonary hyperinflation, which is associated with increased mortality in COPD patients [12]. In cases where PFTs cannot be performed, such as during COVID-19 infection, pulmonary volumetric assessment utilizing quantitative or manual methods on thoracic CT imaging has been proposed as an alternative method to evaluate lung function. This method has been found to correlate with PFT results, irrespective of the presence of fibrosis or emphysema [13]. In our study, the TLCs of the patients were assessed by calculating three distances, namely oblique fissure retraction distance, aortosternal distance, and lung height, through visual CT measurements [14]. COVID-19 radiological severity was assessed using the Total Severity Score (TSS), a standardized measure of the extent of lung involvement in COVID-19 patients [15]. 

Data analysis**

The data of our study were analyzed using IBM SPSS Statistics for Windows, Version 29.0 (Released 2023; IBM Corp., Armonk, New York, United States). For the test of normality, the Shapiro-Wilk test was preferred. Categorical data were presented as numbers and percentages. Continuous data were expressed as mean and standard deviation (SD) if the data were normally distributed; otherwise, medians and interquartile ranges (IQRs) were reported. Continuous variables were analyzed using Student's t-test if they were normally distributed, while non-normally distributed continuous variables were analyzed using the Mann-Whitney U test. Categorical variables were compared using the Chi-Square test or Fisher's exact test, as appropriate. Sequential measurements of dependent measures were compared using a Paired T-test. Univariate and multivariate analyses were used to reveal independent predictors of poor outcomes. Receiver operating characteristic (ROC) analysis was used to calculate the area under the curve (AUC) of the scoring systems. The DeLong and Clarke-Pearson approach was employed to compare the AUC of the scoring systems [16]. Statistical significance was defined as a p-value of less than 0.05.

## Results

Baseline characteristics 

**Figure 1:**
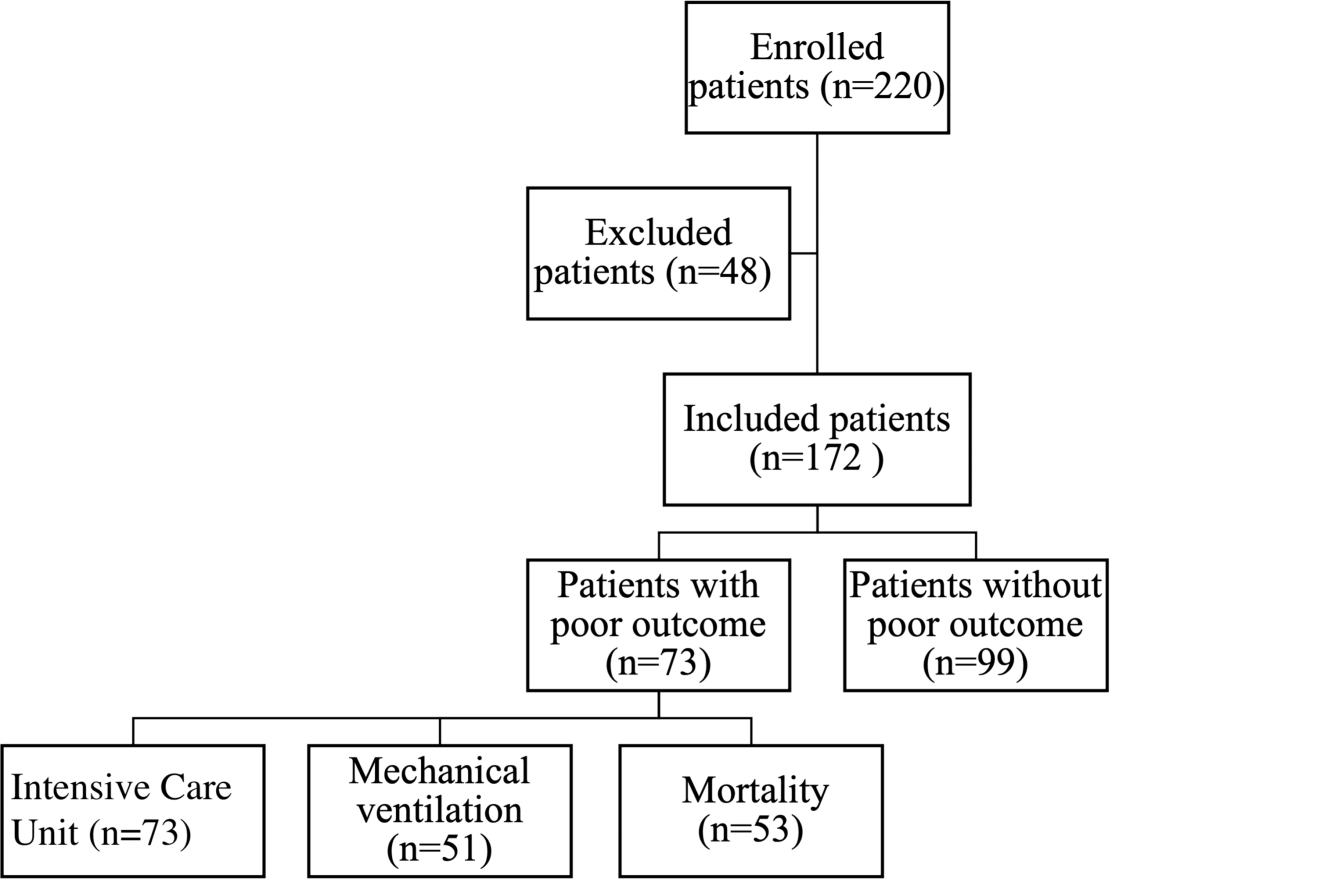
Patient flow chart.

A total of 220 patients admitted to the ED were initially evaluated for eligibility. After applying the exclusion criteria, a final sample of 172 patients was included for statistical analysis. The patient flow chart is presented in Figure 1. The median age of the subjects was 72.5 (66-80) years, with 48 (27.9%) females. A total of 73 (42.4%) patients had a poor outcome during the 30-day follow-up period.

Outcome measures

The incidence of poor outcomes was statistically significantly higher in patients with concomitant chronic kidney disease, decreased diastolic blood pressure, and increased respiratory rate (p=0.009, p=0.004, and p<0.001, respectively). Among the oxygenation parameters, low SpO2, partial pressure of oxygen (PaO2)/FiO2, SpO2/FiO2, and ROX index, as well as high FiO2 were significantly associated with an increased incidence of poor outcome (p<0.001, p=0.005, p<0.001, p<0.001, and p<0.001, respectively). Patients with poor outcomes were more likely to have lower FEV1 and FEV1/forced vital capacity (FVC) on pre-COVID-19 PFT (p=0.001 and p=0.003, respectively), a shorter aortosternal distance measured on thoracic CT at admission, and a higher TSS (p=0.03 and p<0.001, respectively). Laboratory parameters including lymphocytes, fibrinogen, albumin, and C-reactive protein (CRP) were significantly lower in patients with poor outcomes (p=0.003, p=0.02, p=0.009, and p=0.03, respectively). A comparison of descriptive characteristics between the poor outcome groups is presented in Table 1.

**Table 1:** Comparison of demographic, clinical, and laboratory descriptive characteristics between participants with and without poor outcomes *p<0.05 was considered statistically significant. SpO2: peripheral oxygen saturation; PaO2: partial pressure of oxygen; FiO2: fraction of inspired oxygen; ROX: respiratory rate oxygenation; PFT: pulmonary function test; FEV1: forced expiratory volume in one second; FVC: forced vital capacity; WBC: white blood cell; ALT: alanine aminotransferase; AST: aspartate transaminase; BUN: blood urea nitrogen; LDH: lactate dehydrogenase; BNP: B-type natriuretic peptide; CRP: C-reactive protein

Variable	With Poor Outcome (n=73)	Without Poor Outcome (n=99)	p-value
Age (years), median (%25-75 quartiles)	71 (64.5-78.5)	73 (68-82)	0.13
Female gender, n (%)	21 (28.8)	27 (27.3)	0.82
Smoking status, n (%)			0.70
Never smoked	17 (26.6)	28 (30.4)	
Ex-smoking	41 (64.1)	53 (57.6)	
Currently smoking	6 (9.4)	11 (12)	
Cigarettes (pack/year), median (%25-75 quartiles)	50 (40-62.5)	50 (38-60)	0.78
COVID vaccination, n (%)	7 (9.6)	16 (16.2)	0.21
Vital signs			
Fever (°C), mean ± SD	36.9 ± 0.4	36.8 ± 0.4	0.12
Pulse rate (beats/minute), mean ± SD	95.4 ± 18	90.9 ± 17.6	0.10
Systolic blood pressure (mmHg), median (%25-75 quartiles)	124 (110.5-137.5)	129 (120-145)	0.10
Diastolic blood pressure (mmHg), median (%25-75 quartiles)	71 (63-79)	77 (70-85)	0.004
Respiratory rate, median (%25-75 quartiles)	30 (24-35)	20 (18-25)	<0.001
Oxygenation status			
SpO2 (%), mean ± SD	88.5 ± 8.1	92.3 ± 4.3	<0.001
PaO2, median (%25-75 quartiles)	40.4 (30.5–49.7)	41.6 (32-59.9)	0.35
FiO2 (%), mean ± SD	42 ± 30	26 ± 14	<0.001
PaO2/FiO2, median (%25-75 quartiles)	152.3 (68.4-213.8)	175.9 (137.6-261.6)	0.005
SpO2/FiO2, mean ± SD	298.5 ± 138.6	397.8 ± 92.3	<0.001
ROX index, median (%25-75 quartiles)	11.1 (4.7-15.8)	20.1 (14.6-24.6)	<0.001
Comorbidities, n (%)			
Hypertension	55 (75.3)	70 (70.7)	0.50
Diabetes Mellitus	25 (34.2)	37 (37.4)	0.67
Atrial fibrillation	14 (19.2)	19 (19.2)	1.00
Coronary artery disease	37 (50.7)	45 (45.5)	0.49
Chronic heart failure	16 (21.9)	25 (25.3)	0.61
Asthma	35 (47.9)	47 (47.5)	0.95
Chronic kidney disease	15 (20.5)	7 (7.1)	0.009
Cerebrovascular disease	8 (11)	11 (11.1)	0.97
Malignancy	10 (13.7)	14 (14.1)	0.93
PFTs before COVID-19, median (%25-75 quartiles)			
FEV1 (%)	56 (47.5-62.5)	63 (55-68.7)	0.001
FEV1/FVC	61 (55-66.5)	65 (60-68)	0.006
Laboratory parameters			
WBC (10³/UL), mean ± SD	8.25 ± 5.82	7.44 ± 2.98	0.28
Neutrophils (10³/UL), mean ± SD	6.51 ± 4.8	5..52 ± 2.70	0.11
Lymphocytes (10³/UL), mean ± SD	1.00 ± 0.52	1.27 ± 0.58	0.003
Hemoglobin (10³/UL), median (%25-75 quartiles)	12.7 (10.5-14.2)	13 (11.8-14.3)	0.21
Hematocrit (10³/UL), median (%25-75 quartiles)	38.8 (32.9-42.8)	39.7(36.7-43.7)	0.23
Platelets (10³/UL), mean ± SD	213.4 ± 108.4	208.2 ± 68.5	0.72
ALT (u/L), mean ± SD	21.3 ± 11.1	24.0 ± 21.6	0.29
AST (u/L), mean ± SD	38.0 ± 47.5	34.7 ± 26.4	0.56
Albumin (g/L), median (%25-75 quartiles)	35 (32-38.6)	36.8 (34.1-40)	0.02
BUN (mg/dl), mean ± SD	54.4 ± 38.5	54.3 ± 34.8	0.97
Creatinine (mg/dl), mean ± SD	1.26 ± 0.91	1.31 ± 0.97	0.74
LDH (u/L), mean ± SD	379.5 ± 197.6	393.6 ± 172.8	0.70
Fibrinogen (g/dL), mean ± SD	507.3 ± 140.9	610.2 ± 213.8	0.009
D-dimer (ng/mL), mean ± SD	1401.9± 1149.8	2214.0± 3091.4	0.13
Troponin (ng/dL), mean ± SD	0.05 ± 0.14	0.04 ± 0.05	0.47
BNP (pg/mL), mean ± SD	2627.0 ± 5092.1	2436.9 ± 6141.6	0.89
CRP (mg/L), median (%25-75 quartiles)	40 (11-90)	45 (20-123)	0.16
Visual tomographic measurements of total lung capacity			
Aortosternal distance (mm), mean ± SD	28.0 ± 8.2	31.0 ± 9.1	0.03
Oblique fissure retraction distance, mean ± SD	0.65 ± 0.2	0.66 ± 0.44	0.90
Lung height, mean ± SD	16.9 ± 5.7	17.6 ± 9.1	0.55
COVID-19 radiological severity score, mean ± SD	7.40 ± 6.2	2.9 ± 3.5	<0.001
Long-term oxygen therapy, n (%)	14 (19.2)	17 (17.2)	0.73

The variables that exhibited significant differences between the groups regarding the primary outcome were included in the multivariate analysis using a logistic regression model. SpO2, FiO2, and respiratory rate, which constitute the ROX index, were not included in the multivariate analysis. The variables albumin and FEV1/FVC were not included in the multivariate analysis due to their lack of clinical significance. Similarly, fibrinogen was excluded from the analysis due to a high percentage of missing data (>20%). Low ROX index and FEV1 and high TSS were found to be statistically significantly associated with poor outcomes in multivariate analysis (p<0.001, p=0.004, and p=0.004, respectively) (Table 2).

**Table 2:** Results of multivariate logistic regression analysis of independent predictors of poor outcomes. *p<0.05 was considered statistically significant. The goodness of fit was confirmed by the Hosmer-Lemeshow test (p = 0.135) ROX: The respiratory rate oxygenation, FEV1: forced expiratory volume in one second

	Odds ratio (OR)	Confidence interval (CI) (95%)	p-value
Diastolic blood pressure	0.975	0.944- 1.006	0.11
ROX index	0.867	0.814- 0.925	<0.001
Lymphocyte count	0.520	0.237 – 1.14	0.10
Chronic kidney disease	1.892	0.521- 6.878	0.33
FEV1	0.949	0.916- 0.984	0.004
COVID-19 radiological severity score	1.15	1.045- 1.266	0.004
Aortosternal distance	0.977	0.930- 1.027	0.36
Constant	5,527		0.004

**Figure 2:**
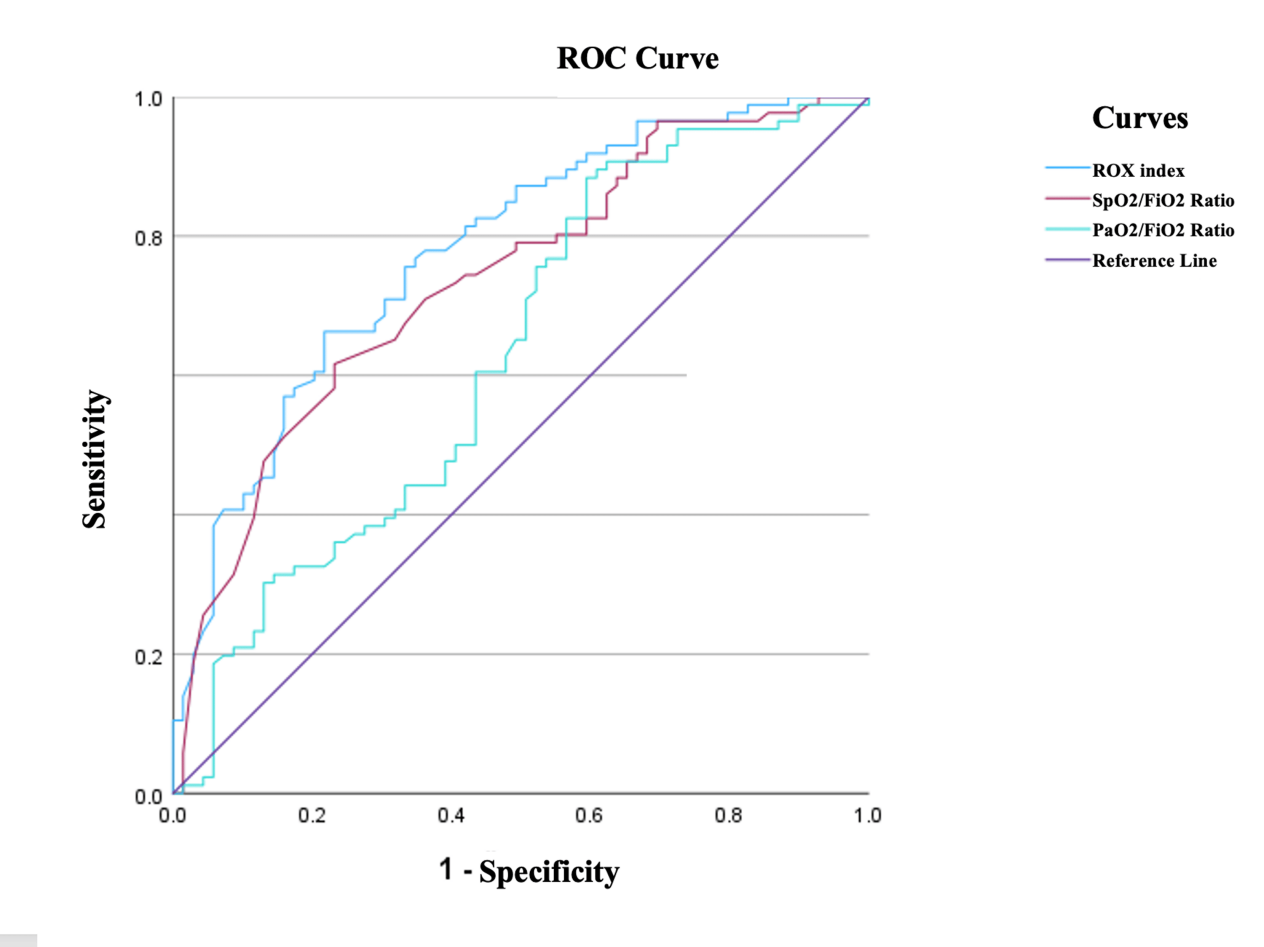
ROC analysis of ROX index, SpO2/FiO2 ratio, and PaO2/FiO2 ratio in predicting poor outcomes. ROC: receiver operating characteristic; ROX: respiratory rate oxygenation; SpO2: peripheral oxygen saturation; FiO2: fraction of inspired oxygen; PaO2: partial pressure of oxygen

The diagnostic value of the TSS and ROX index in predicting the 30-day poor outcome was analyzed by the ROC curve and the AUC was calculated as 0.71 (95%CI: 0.638-0.796, p<0.001) and 0.80 (95%CI= 0.730-0.863, p<0.001), respectively. Regarding the oxygenation status, the associations of ROX index, SpO2/FiO2 ratio, and PaO2/FiO2 ratio with poor outcome were evaluated using ROC analysis, and the AUC was calculated as 0.80 (95%CI= 0.730-0.863), 0.737 (95%CI= 0.659-0.815) and 0.631 (95%CI= 0.541-0.541), respectively (Figure 2). 

When comparing the AUC values using the DeLong test, the ROX index was found to be a better predictor of poor outcome than SpO2/FiO2 and PaO2/FiO2 ratio (AUC=0.80 vs. 0.73, p=0.01; AUC= 0. 80 vs. 0.63 p=0.001, respectively). The diagnostic test performance of the parameters in predicting the 30-day poor outcome is presented in Table 3.

**Table 3:** Diagnostic test performance results of, ROX index, SpO2/fiO2 ratio, PaO2/FiO2 ratio, and COVID-19 radiologic severity score in predicting poor outcome ^a^threshold 16.4; ^b^threshold 421.4; ^c^threshold 104.8; ^d^threshold 4.5 CI: confidence interval; AUC: area under the curve; ROX: respiratory rate oxygenation; SpO2: peripheral oxygen saturation; PaO2: partial pressure of oxygen; FiO2: fraction of inspired oxygen

Criterion, (95% CI)	ROX index ^a^	SpO2/FiO2 ^b^	PaO2/FiO2 ratio ^c^	COVID-19 radiologic severity score ^d^
AUC	0.80 (0.730–0.863)	0.737 (0.659–0.815)	0.631(0.541–0.721)	0.72 (0.64 – 0.80)
Sensitivity (%)	68.6 (58.5–77.6)	72.5 (60.4 – 82.5)	72.5 (60.4 – 82.5)	58.9% (46.7- 70.2)
Specificity (%)	78.0 (66.8–86.9)	74.4 (63.9- 83.2)	76.7 (66.4 – 85.2)	74.2% (64.3-82.5)
Positive Likelihood Ratio	2.4 (1.8 – 3.4)	2.8 (1.9 – 4.2)	3.1 (2.1 – 4.7)	2.2 (1.5- 3.3)
Negative Likelihood Ratio	0.3 (0.2 – 0.5)	0.4 (0.3 – 0.6)	0.4 (0.2 – 0.5)	0.5 (0,4- 0.7)
Positive Predictive Value (%)	64.7 (57.2–71.6)	69.4 (60.6 – 77)	71.4 (62.4 – 79)	63.2% (53.8- 71.7)
Negative Predictive Value (%)	80.9 (72.9–86.9)	77.1 (69.3 – 83.4)	77.7 (70 – 83.8)	70,5% (64.0-76.3)
Accuracy	72.6 (65.3–79.1)	73.6 (65.9 – 80.3)	74.8 (67.3 – 81.5)	67.6% (60.0- 74.6)

**Figure 3:**
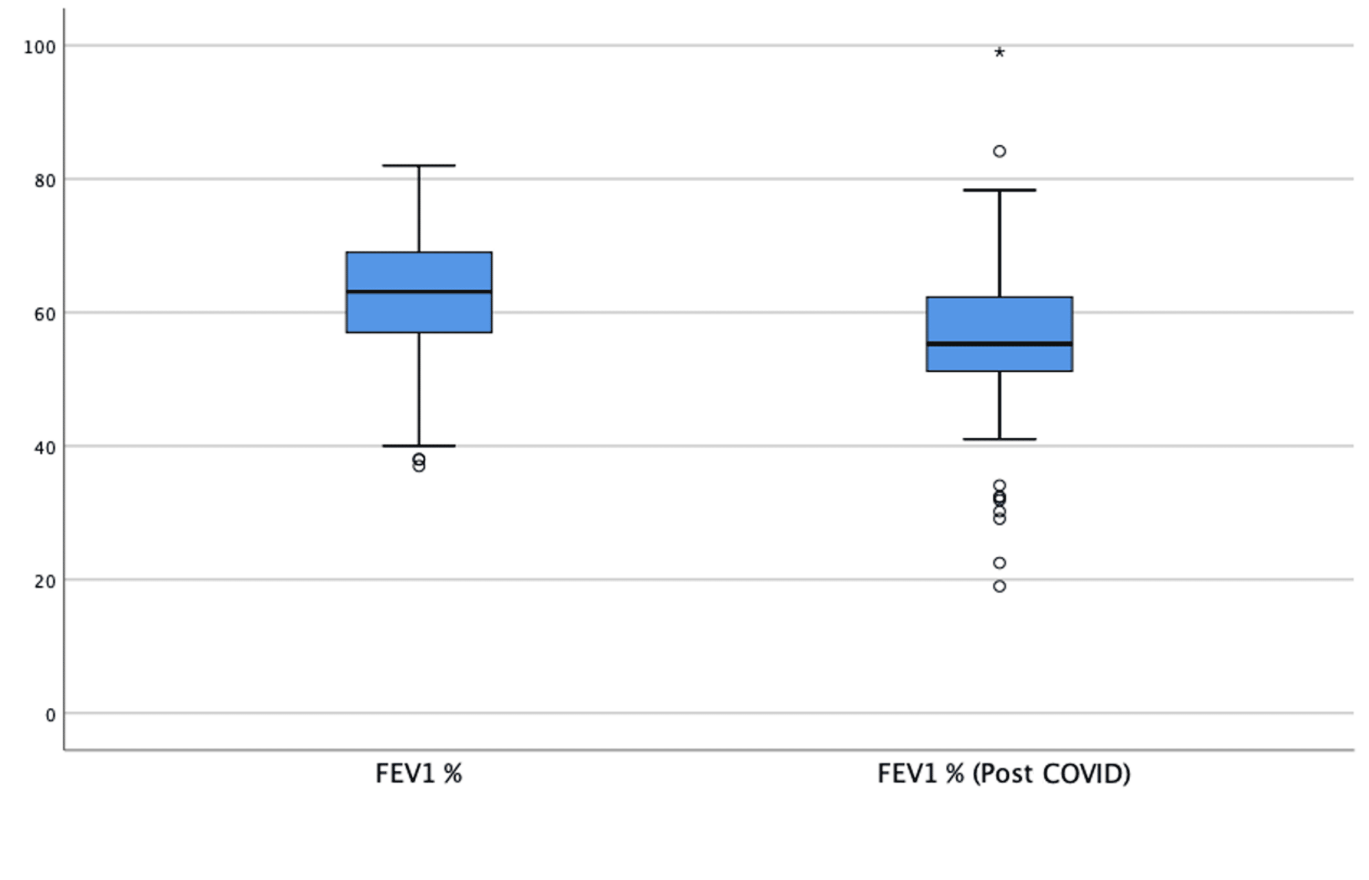
Comparison of expected values of FEV1 before and after COVID-19. FEV1: forced expiratory volume in one second

Among the poor outcome negative patients (n=99), eight patients dropped out of outpatient follow-up and 10 patients failed to complete PFT, leaving 81 patients who successfully underwent PFT following COVID-19. A significant decrease in FEV1 was observed after COVID-19 compared to baseline values (post-COVID-19 FEV1% = 55.9 ± 12.9; baseline FEV1 = 62.1 ± 10.0; p<0.001). The distribution of patients according to GOLD stages was as follows: four patients (2.3%) were in stage 1, 122 patients (70.9%) in stage 2, 42 patients (24.4%) in stage 3, and four patients (2.3%) in stage 4. The comparison of FEV1 values before and after COVID-19 is shown in Figure 3. 

In the analysis of factors associated with readmission within 14 days due to respiratory symptoms in all discharged patients (n=119), irrespective of the outcome group, it was observed that the presence of coronary artery disease (CAD) significantly increased the frequency of readmission within 14 days (OR:2.66, 95%CI: 1.13-6.27, p =0.022).

## Discussion

Our research findings establish that for COPD patients with COVID-19 who presented to the ED, low ROX index, low baseline FEV1 value, and high TSS score were independently associated with an increased risk of poor outcome within 30 days. Significant decreases in FEV1 values were noted during the third month after COVID-19 infection in patients who did not experience poor outcomes, as compared to their baseline FEV1 values. Additionally, among COPD patients who were discharged, those with co-existing CAD were more likely to be readmitted due to respiratory symptoms.

ROX index is a non-invasive tool that uses variables related to oxygenation and respiratory failure. It has proven useful in assessing poor prognosis and intubation risk in COVID-19 pneumonia patients [17]. In a study analyzing 554 patients admitted to the ED due to COVID-19, a ROX index <22.3 was found to be significantly associated with an increased incidence of 30-day mortality (AUC = 0.764, 95%CI 0.708-0.820, p < 0.001) [18]. Impairment of oxygenation is recognized in both COVID-19 pneumonia and COPD patients, and it is hypothesized that the impairment may be more prominent when these two conditions coexist. However, there is limited data on this patient group. In our study, we found that a ROX index <16.4 in COPD patients with COVID-19 was significantly associated with a higher incidence of poor outcomes (AUC=0.80, 95%CI: 0.730-0.863, p<0.001). The observed lower threshold value of the ROX index in our study compared to the literature suggests that COPD exacerbates the underlying hypoxia caused by COVID-19 pneumonia. This is further supported by the finding of a low median ROX index among the patients in our study.

It is well-established that the PaO2/FiO2 and SpO2/FiO2 ratios exhibit strong predictive ability for severe disease and mortality (AUC=0.918 and 0.901, respectively). The SpO2/FiO2 ratio has gained recognition as a noninvasive and quickly obtainable alternative for assessing oxygenation status [19]. However, it has been shown that the ROX index alone has a greater predictive value compared to both SpO2/FiO2 and respiratory rate alone [20]. In our study, the ROX index was found to perform better than the SpO2/FiO2 ratio and PaO2/FiO2 ratio in predicting the poor outcome. Therefore, we conclude that the ROX index is the most valuable parameter for monitoring oxygenation in COPD patients with COVID-19.

Our study had a significant association between low baseline FEV1 levels and an increased incidence of 30-day poor outcomes (OR: 0.949, 95%CI: 0.916-0.984, p=0.001). Nevertheless, it should be noted that the effect size was not substantial. This could be attributed to the fact that the majority of patients included had moderate to high FEV1 values. It is plausible that the impact of low baseline FEV1 levels on outcomes might be more pronounced in the moderate and severe COPD patient subgroups. However, the available data in our study were inadequate for conducting a subgroup analysis to test this hypothesis. Antúnez et al. conducted a subgroup analysis involving 364 COPD patients with COVID-19, where the majority of patients (71.2%) were classified as GOLD stages 1 and 2 [21]. Notably, their study did not find any significant association between low FEV1 values and mortality. In our study, due to the limited number of patients in GOLD stages 1 and 4, we opted to evaluate FEV1 values without categorization.

In the study by Kunwei et al. investigating the relationship between radiologic findings and clinical classification of COVID-19, the TSS score demonstrated a high predictive value for severe disease, with an AUC of 0.918 (95%CI 0.843-0.994). Using a threshold value of 7.5, the TSS exhibited a sensitivity of 82.6% and a specificity of 100% [22]. Additionally, a meta-analysis by Zakariaee et al. revealed that the COVID-19 radiologic severity score was an independent predictor of mortality, (OR: 7.12, 95%CI: 5.31-9.56) [23]. In our analyses, we also observed that the TSS served as an independent predictor of poor outcomes in the COPD subgroup (AUC: 0.71, 95%CI: 0.63-0.79, p<0.001). 

A significant decline in mean FEV1 values was observed in the current study among patients in the post-COVID-19 period compared to their baseline values. Similar findings have been reported in the literature, indicating a decline in PFT values in the long term after COVID-19 in patients without a pre-existing diagnosis of COPD [7]. Limited information is available in the literature regarding the long-term pulmonary function changes in COPD patients with COVID-19. Therefore, the findings of our study hold significant value.

Limitations

The single-center nature of our study introduces the possibility that the sample may represent a limited population, thus limiting the generalizability of the findings. Moreover, we acknowledge that the sample size calculated for the study's primary endpoint may not have been adequate to assess the secondary endpoints comprehensively. We recorded baseline PFTs within the last year before admission; however, it is possible that there were factors contributing to the decline in FEV1 during this period before the development of COVID-19. Also, we did not consider the treatment data, which could also affect the outcome. In our study, patient enrollment began on December 1, 2020, while COVID-19 vaccination in our country started on January 13, 2021. Given that vaccination status can potentially influence primary and secondary outcomes, the low percentage of vaccinated patients in our study may have led to a type-2 error in this patient group. Our study was also limited by the fact that a poor outcome was defined as admission to the ICU or mortality due to any cause.

## Conclusions

Baseline FEV1, COVID-19 radiological severity score, and ROX index were independent predictors of poor prognosis in COPD patients admitted to the ED due to COVID-19. Among patients without a poor outcome, a significant decline in FEV1 values was observed in the third month post-COVID-19 compared to baseline values. In discharged patients, the presence of CAD was found to be a risk factor predicting readmission with respiratory symptoms
